# Characterization of the *pgf* operon involved in the posttranslational modification of *Streptococcus mutans* surface proteins

**DOI:** 10.1038/s41598-018-23170-3

**Published:** 2018-03-16

**Authors:** Alejandro Avilés-Reyes, Irlan Almeida Freires, Richard Besingi, Sangeetha Purushotham, Champion Deivanayagam, L. Jeannine Brady, Jacqueline Abranches, José A. Lemos

**Affiliations:** 1 Department of Oral Biology, University of Florida, College of Dentistry, Gainesville, FL USA; 2 Department of Biochemistry and Molecular Genetics, University of Alabama, Birmingham, AL USA

## Abstract

Protein glycosylation has been described as the most abundant and complex post-translational modification occurring in nature. Recent studies have enhanced our view of how this modification occurs in bacteria highlighting the role of protein glycosylation in various processes such as biofilm formation, virulence and host-microbe interactions. We recently showed that the collagen- and laminin-binding adhesin Cnm of the dental pathogen *Streptococcus mutans* is post-translationally modified by the PgfS glycosyltransferase. Following this initial identification of Cnm as a glycoprotein, we have now identified additional genes (*pgfM1*, *pgfE* and *pgfM2*) that are also involved in the posttranslational modification of Cnm. Similar to the previously characterized Δ*pgfS* strain, inactivation of *pgfM1*, *pgfE* or *pgfM2* directly impacts Cnm by altering its migration pattern, proteolytic stability and function. In addition, we identified the wall-associated protein A (WapA) as an additional substrate of Pgf-dependent modification. We conclude that the *pgS-pgfM1-pgfE-pgfM2* operon encodes for a protein machinery that can modify, likely through the addition of glycans, both core and non-core gene products in *S*. *mutans*.

## Introduction

Protein glycosylation is considered the most abundant form of post-translational modification among living organisms and its broad distribution indicates that it is advantageous to the overall fitness of both prokaryotes and eukaryotes^1,2^. In bacteria, glycan decoration of surface-associated or secreted proteins has been shown to modulate an array of cellular processes, such as migration, communication and adhesion^1,3–7^. There are two major types of protein glycosylation; *N*-glycosylation and *O*-glycosylation whereby sugar moieties are directly attached to the amino terminal of an asparagine (*N*-glycosylation) or to the hydroxyl group of a serine or a threonine (*O*-glycosylation)^1,8^. First described in the gastrointestinal pathogen *Campylobacter jejuni*, bacterial *N*-glycosylation relies on the general Pgl glycosylation pathway, which is found in a large number of Gram-negative organisms^9^. Bacterial *O*-glycosylation, on the other hand, is far less characterized as the identification of specific *O*-glycosyltransferases and *O*-oligosaccharyltransferases is much more difficult due to their high level of heterogeneity compared to the canonical *N*-glycosylation machinery^8,10^. The complete characterization of *O*-glycosylated proteins is also more difficult as the system is generally more complex and often relies on stepwise attachment of glycoconjugates^1^. Given that bacterial glycoproteins are of increasing interest due to their abundance in nature and importance in health and disease, the analysis and characterization of glycosylation patterns is of great importance as it could represent new avenues of basic and clinical research^11^.

Sugar metabolism plays an essential role in the biology of the dental pathogen *Streptococcus mutans* contributing to energy generation, gene regulation, biofilm formation and virulence^12–15^. However, the use of sugar moieties to post-translationally modify proteins in *S*. *mutans* is poorly characterized. Recently, we showed that the collagen- and laminin-binding protein Cnm of *S*. *mutans* is post-translationally modified and that such modification requires the expression of a gene located downstream and co-transcribed with *cnm* that we named *pgfS* (for protein glycosyltransferase of *S*. *mutans*)^6^. Of note, the *cnm* gene is present in approximately 15% of *S*. *mutans* isolates and encodes an important virulence factor that mediates intracellular invasion and has been associated with systemic infections and caries severity^16–24^. Loss of PgfS-dependent modification of Cnm led to decreased collagen-binding activity and decreased resistance to protease degradation suggesting that Cnm glycosylation is required for both protein function and stability^6^. Immediately downstream and co-translated with *pgfS* is a gene encoding a putative integral membrane protein (*smu2066c*) that is followed by genes encoding an UDP-4-glucose epimerase (*smu2065c*) and a second membrane protein (*smu2064c*). Considering that bacterial protein glycosylation machineries often localize at the intracellular membrane interface^1^, the proximity of the glycosyltransferase *pgfS* gene with genes encoding a sugar epimerase and two membrane proteins suggested that together these four gene products represent an uncharacterized glycosylation system. Using Cnm as a model protein, we show herein that *smu2064c*, *smu2065c* and *smu2066c* are indeed required for the post-translational modification of Cnm. In many cases, strains lacking any one of these genes phenocopied the Δ*pgfS* strain although in the *smu2064c* deletion mutant Cnm appears to undergo partial modification. Interestingly, *pgfS*, *smu2064c*, *smu2065c* and *smu2066c* are found in all *S*. *mutans* strains sequenced to date and are therefore part of the *S*. *mutans* core genome. This observation led us to hypothesize that the role of these genes in protein decoration extends beyond Cnm glycosylation. In fact, we found that the surface adhesin WapA, encoded within the *S*. *mutans* core genome, is also modified by the Pgf system. Thus, we conclude that the *pgfS-smu2064c*-*smu2065c*-*smu2066c* gene cluster represents a novel posttranslational modification machinery, exerting its function likely through O-glycosylation, of *S*. *mutans* capable of modifying both core and non-core proteins.

## Results

### Transcriptional organization of the *pgf* genes

**Figure 1:**
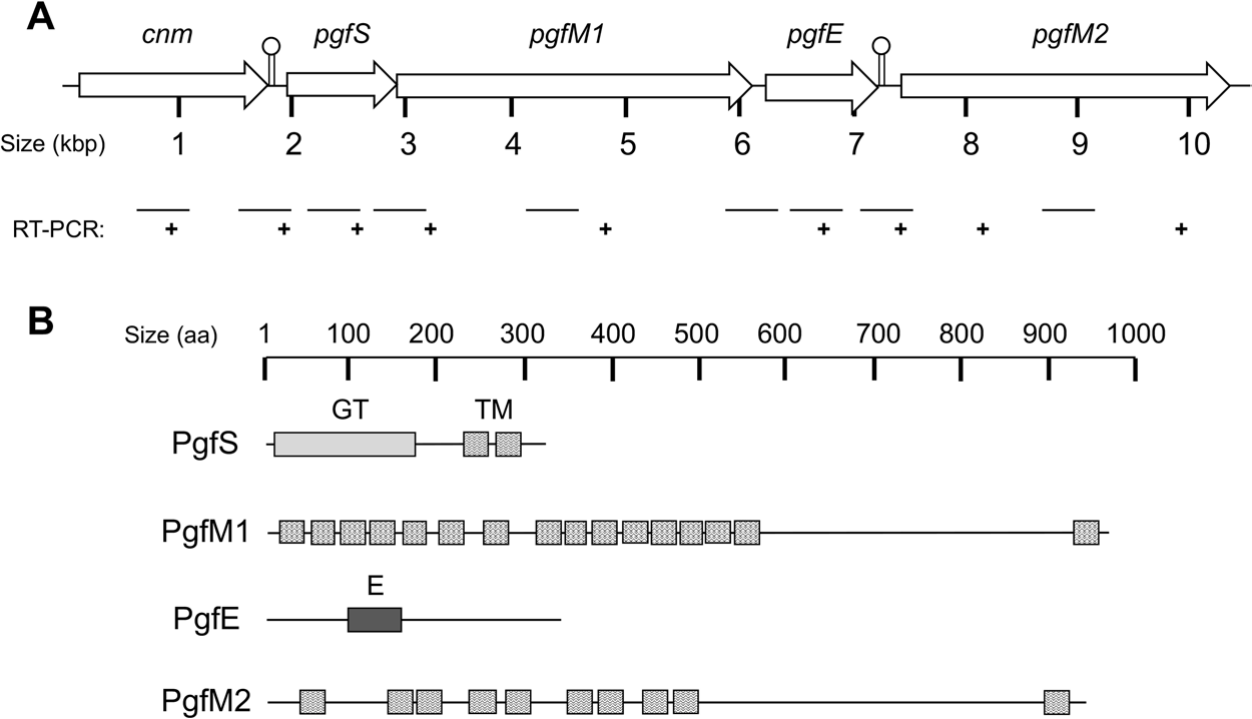
Genetic organization of the *pgf* operon in *S*. *mutans* OMZ175 and conserved domains of Pgf proteins. (**A**) Despite the presence of putative Rho-independent transcriptional terminators (indicated by lollypop symbols) between *cnm*-*pgfS* and *pgfE*-*pgfM2*, RT-PCR analysis revealed that *cnm*, *pgfS*, *pgfM1* and *pgfE* are co-transcribed. (**B**) Predicted domain organization of PgfS, PgfM1, PgfE and PgfM2: (GT) glycosyltransferase enzymatic domain, (TM) transmembrane domain, (**E**) epimerase enzymatic domain.

Previously, we demonstrated the requirement of *pgfS*, encoding a membrane-bound GT-A type glycosyltransferase, in the post-translational modification of Cnm^6^. Bioinformatics analysis of the available genome sequences of *S*. *mutans* revealed that *pgfS* is transcriptionally-coupled with *smu2066c* (herein *pgfM1*), which encodes a membrane protein of the *O*-mannosyltransferase (PMT)-2 subfamily containing 16 putative trans-membrane (TM) domains (Fig. 1A and B). While the protein product of *S*. *mutans pgfM1* is not predicted to possess enzymatic activity, eukaryotic members of this family have been shown to catalyze the transfer of mannose from dolichyl phosphate-activated mannose (Dol-P-Man) to serine or threonine residues of secretory proteins^25^. Downstream of *smu2066c* and separated by 35 base pairs (bp) is *smu2065c* (herein *pgfE*), which encodes a UDP-4-glucose epimerase predicted to catalyze the interconversion of UDP-glucose and UDP-galactose. Further downstream and separated by 133 bp, *smu2064c* (herein *pgfM2*) encodes another membrane protein, with 10 putative TM domains, that shares 54% homology with PgfM1 (Fig. 1B).

RT-PCR analyses revealed that *pgfS*, *pgfM1* and *pgfE* are co-transcribed (Figs 1A and S1). These results are in agreement with *in silico* analysis that indicates that there are no putative promoters or transcriptional terminators in the 3′ UTR between *pgfM1* and *pgfE*. On the other hand, the 133-bp intergenic region between *pgfE* and *pgfM2* contains a putative Rho-independent transcriptional terminator located 4 bp downstream of the *pgfE* stop codon, which is followed by a putative σ^A^-type promoter located 86 bp upstream of the *pgfM2* start codon. However, RT-PCR analysis indicated that *pgfE* and *pgfM2* are also co-transcribed possibly because transcription termination at *pgfE* is not 100% efficient, which is similar to the terminator located between *cnm* and *pgfS*^6^. Finally, RT-PCR analysis revealed that the gene downstream *pgfM2*, which encodes a putative ferrochelatase, is not co-transcribed with *pgfM2* (data not shown).

### All genes within the *pgf* operon contribute to modification and stability of Cnm

**Figure 2:**
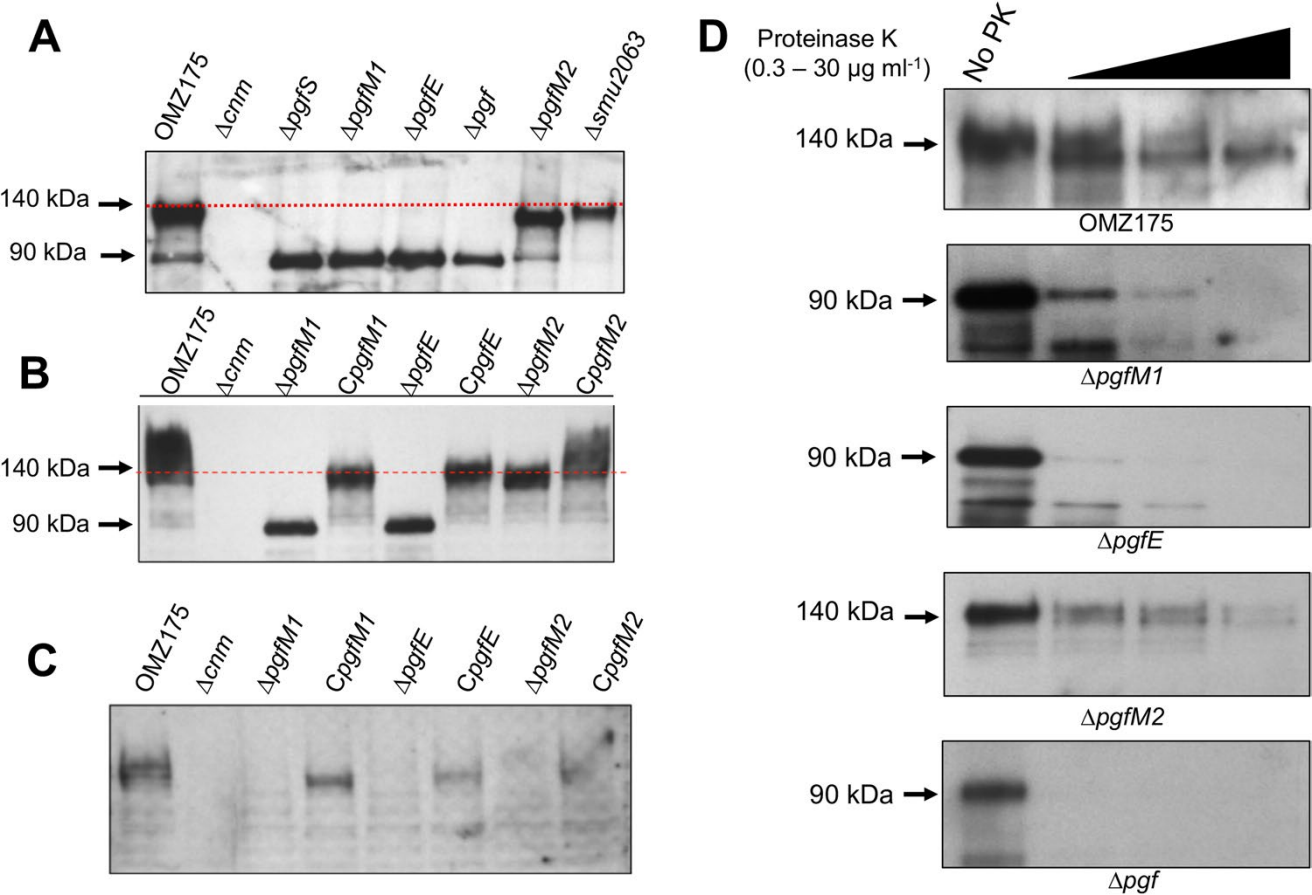
Modification of Cnm by the *pgf* operon. (**A**) Western blot analysis using anti-rCnm shows a band of ~140 kDa corresponding to the fully mature Cnm in OMZ175. Smaller variants of Cnm correspond to partially glycosylated (~120 kDa, Δ*pgfM2*) and unglycosylated Cnm (~90 kDa, Δ*pgfS*, Δ*pgfM1*, Δ*pgfE* and Δ*pgf*). (**B**) In locus complementation of the single mutants restored the size of Cnm. (**C**) Lectin-binding analysis using biotinylated wheat germ agglutinin (WGA) shows that the mature Cnm produced by OMZ175 is recognized by WGA whereas the Cnm versions produced by the different *pgf* mutans are not. (**D**) Whole cells of strains OMZ175 and Δ*pgf* derivatives were treated with increasing concentrations of proteinase K for 30 min and Cnm stability monitored by Western blotting. In all cases, duplicated gels stained with Coomasie blue were used to confirm that the same amounts of protein were loaded to the individual lanes.

To assess the involvement of *pgfM1*, *pgfE* and *pgfM2* in the post-translational modification of Cnm, strains bearing individual gene deletions were generated (Δ*pgfM1*, Δ*pgfE* and Δ*pgfM2*) as well as a quadruple mutant lacking all four *pgf* genes (Δ*pgf*). All mutant strains were readily isolated and grew as well as the parent strain OMZ175 under balanced growth conditions (i.e., BHI at 37 °C in a 5% CO_2_ atmosphere; data not shown). However, Western blot analysis revealed that the mobility of Cnm was altered in all mutants compared to the parent strain OMZ175 (Fig. 2A), a trait that could be restored after reintroduction of each deleted *pgf* gene (Fig. 2B). Specifically, similar to the Δ*pgfS* strain^6^, deletion of *pgfM1*, *pgfE* or the entire *pgf* operon produced a ~90 kDa variant of Cnm that was substantially smaller than the 140 kDa product identified in OMZ175. On the other hand, deletion of *pgfM2* yielded an intermediate-sized protein band of ~120 kDa. Finally, Cnm migration on SDS-PAGE was not altered in a strain lacking the ferrochetalase-encoding *smu2063c* gene, which lies downstream of the *pgf* operon (Fig. 2A). Because *smu2063c* is not co-transcribed with *pgfM2 and* does not modify Cnm, we did not include this strain in subsequent experiments. It is worth mentioning that we have previously indicated that the mature Cnm migrates at ~120 kDa^6,19^. However, increased resolution of migration on SDS-PAGE using 10% acrylamide gels identified Cnm migrating at ~140 kDa. Moreover, Cnm has a predicted molecular weight of ~54 kDa, indicating that Cnm has an abnormal electrophoretic migration pattern even in the absence of Pgf modification^6^. Whole protein analysis of purified native Cnm by quadruple time of flight (Q-TOF) mass spectrometry confirmed the predicted 54 kDa product (data not shown). Thus, the aberrant migration of Cnm in SDS-PAGE is likely due to the combination of Pgf-dependent glycosylation as well as the high negative charge density of the Cnm C-terminal domain as discussed previously^6,19^.

In agreement with the notion that the Pgf system is responsible for Cnm glycosylation, reactivity to the WGA lectin (was lost in all mutants but restored in the complemented strains (Fig. 2C). Of note, we previously showed that Cnm interacts with WGA but not with other lectins such as PNA and ConA^6^. In addition, we showed that Cnm also interacts with succinylated WGA, which recognizes only *N*-acetylglucosamine and that this interaction is inhibited by *N*-acetylglucosamine in a concentration-dependent manner^6^. Collectively, these results strongly suggest that PgfM1, PgfE and PgfM2 act in concert with PgfS to glycosylate Cnm. In addition, the intermediate size of Cnm in the Δ*pgfM2* strain indicates that Cnm can be modified in the absence of PgfM2. The lack of WGA reactivity in the Δ*pgfM2* mutant suggests that PgfM2 is responsible for the addition of the *N*-acetylglucosamine, the sugar moiety recognized by WGA in the lection blot^6^.

In a previous study, we also showed that the unmodified Cnm version produced by the Δ*pgfS* strain was highly susceptible to protease degradation^6^. Thus, we also expected the Cnm versions produced by the Δ*pgfE*, Δ*pgfM1* and Δ*pgf* strains to be less stable than the mature Cnm version. In fact, inactivation of *pgfM1*, *pgfE* or the entire *pgf* operon increased the susceptibility of Cnm to proteinase K compared to the fully glycosylated Cnm produced by the parent strain OMZ175 (Fig. 2D). In particular, Cnm was completely degraded by as little as 0.3 μg ml^−1^ of proteinase K in the quadruple Δ*pgf* mutant. Consistent with the idea that Cnm is partially glycosylated in the Δ*pgfM2* mutant, the Cnm product observed in this strain was slightly more resistant to proteinase K degradation than the Cnm products of the other Δ*pgf* strains. It was, however, still more susceptible than that produced by OMZ175 (Fig. 2D).

### Cnm-dependent phenotypes are negatively affected in Δ*pgf* mutants

**Figure 3:**
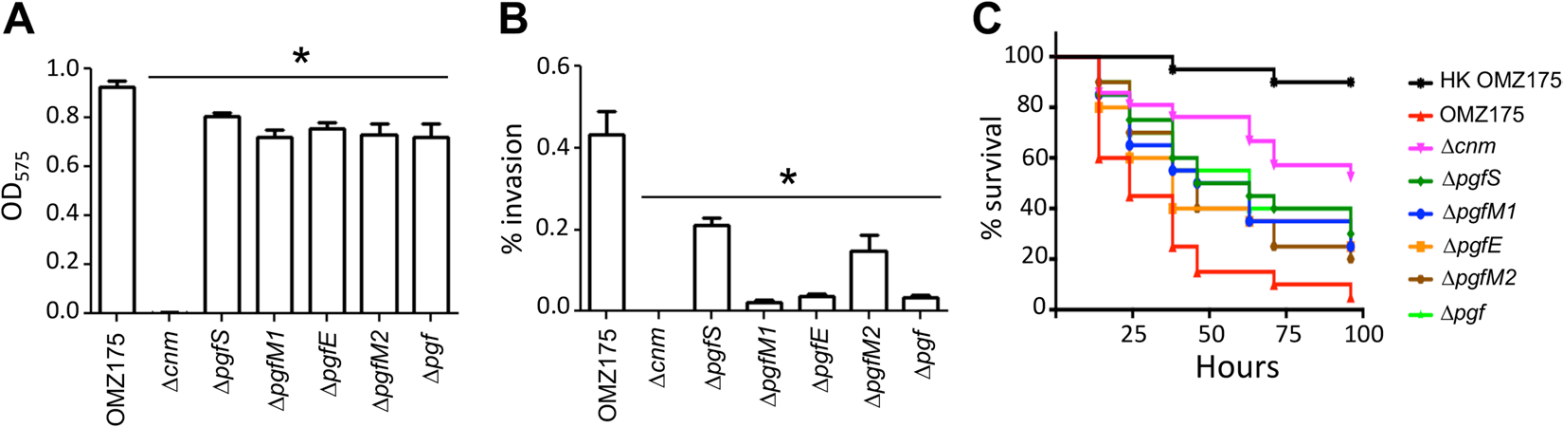
Phenotypic characterization of Δ*pgf* strains. (**A**) Collagen binding of *S*. *mutans* OMZ175 and its Δ*pgf* derivatives. (**B**) Antibiotic protection assay showing the percentage of HCAEC invasion for each strain of *S*. *mutans* after 5 h of incubation. *P ≤ 0.05. (**C**) Killing of *G*. *mellonella* larvae infected with *S*. *mutans* strains over a 96 h period. When compared to the parent OMZ175, virulence of all mutant strains was significantly attenuated (*P* ≤ 0.05).

Expression of Cnm has been linked to robust collagen binding, human coronary artery endothelial cell (HCAEC) invasion and increased virulence in the *Galleria mellonella* invertebrate model^18,19^. Based on the defects in collagen binding, HCAEC invasion and killing of *G*. *mellonella* observed for the Δ*pgfS* strain^6^, we asked if Δ*pgfE*, Δ*pgfM1* and, perhaps, Δ*pgfM2* can phenocopy the Δ*pgfS* strain. A small but significant decrease in collagen binding (~20%) was observed for each *pgf* mutant (Fig. 3A) and HCAEC invasion rates were significantly reduced in the different *pgf* mutant backgrounds, particularly in the Δ*pgfE*, Δ*pgfM1* and Δ*pgf* strains (~90%) (Fig. 3B). Finally, mortality rates of *G*. *mellonella* were significantly lower (*p* < 0.05) in larvae infected with the *pgf* mutant strains indicating that loss of Cnm posttranslational modification negatively impacts *S*. *mutans* virulence (Fig. 3C).

### Cnm modification does not contribute to collagen-binding activity

**Figure 4:**
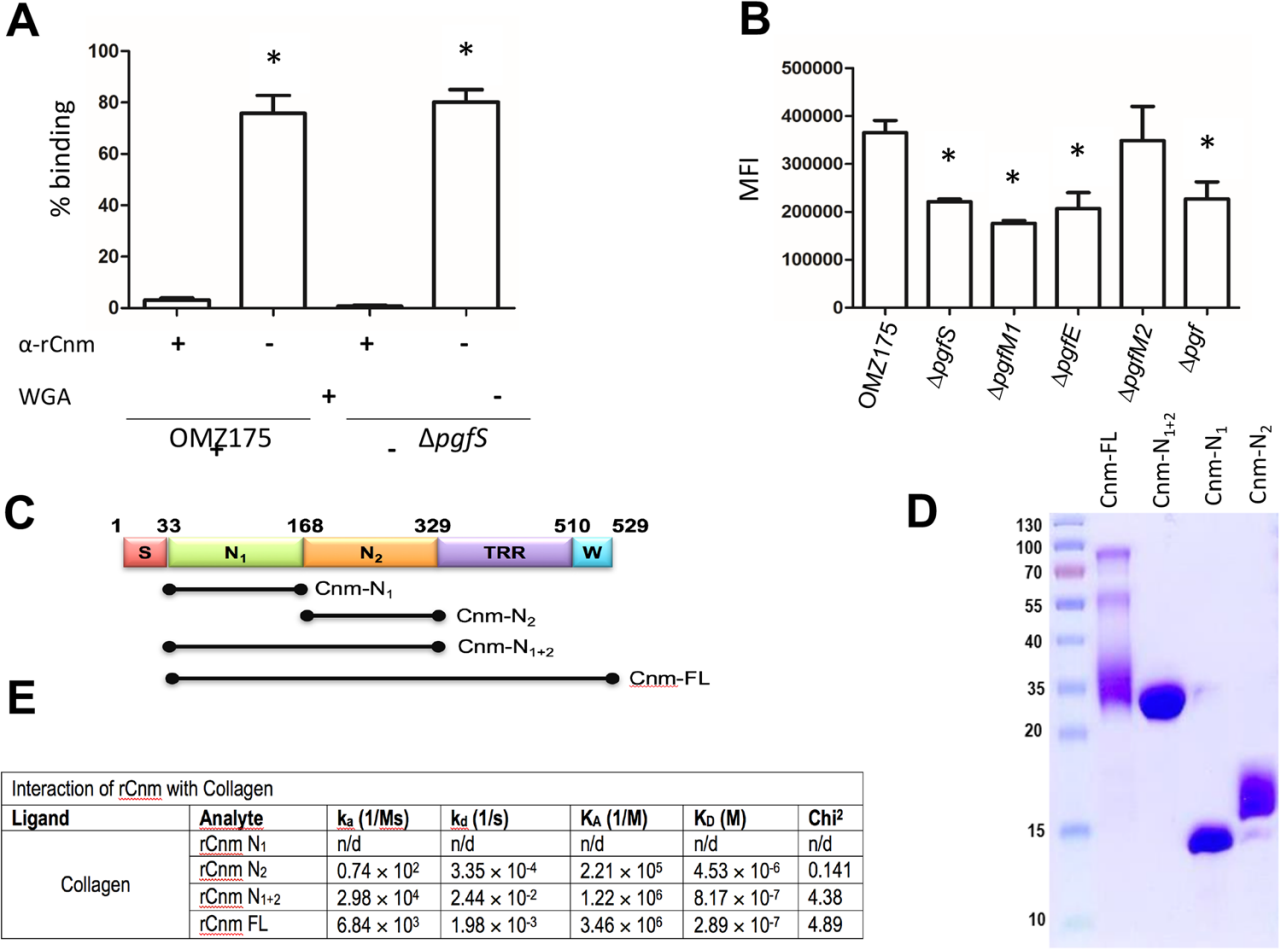
Cnm does not depend on posttranslational modification for collagen recognition. (**A**) Inhibition of collagen binding by anti-Cnm and WGA. (**B**) Expression of Cnm on the surface of OMZ175 and its Δ*pgf* derivatives by flow cytometry expressed as mean fluorescence intensity (MFI). (**C** and **D**) Different Cnm constructs used in SPR analysis. The different Cnm domains shown in panel C are: (S) secretion signal, (N1) collagen-binding subdomain N1, (N2) collagen-binding subdomain N2, (TRR) threonine-rich region, and (W) LPxTG motif. (**E**) SPR analysis of the Cnm constructs. The constructs were injected at concentration of 0.125 μM, 0.25 μM, 0.5 μM, 1.0 μM and 2.0 μM collagen-prepared chip surfaces and the kinetics of association (K_A_) and dissociation (K_D_) rate constants (**C**) were deduced using the 1:1 Langmuir Kinetic model. All experiments were carried out at least in triplicate.

At this point, it remains unclear if posttranslational modification is directly important for Cnm function, or if the phenotypes observed are solely linked to the observed loss of protein stability. To begin to address this question we tested the effect of WGA, which is predicted to interact with the glycosylated threonine-rich region (TRR) of Cnm, on collagen-binding activity of OMZ175 and Δ*pgf* strains. We found that WGA has a modest effect (∼20% inhibition) on the collagen-binding activity of either OMZ175 or Δ*pgfS* (Fig. 4A). However, WGA interfered with the collagen binding capacity of glycosylated (from OMZ175) or unglycosylated (from Δ*pgfS*) Cnm in a similar manner suggesting that the main function of Cnm modification is to increase protein stability. As seen before^19^, a polyclonal antibody against the Cnm collagen-binding domain (CBD) and upstream N-terminus, which are not predicted to undergo post-translational modification, nearly abolished the ability of OMZ175 and Δ*pgfS* to bind to collagen (Fig. 4A). Next, we used flow cytometry to evaluate the reactivity of anti-Cnm antibodies with the bacterial cell surface. When compared to OMZ175, the mean fluorescence intensity (MFI) of Cnm was significantly lower in the *pgf* mutants, except Δ*pgfM2* (Figs 4B and S2). These results are consistent with the diminished stability of Cnm produced by these strains, although we cannot rule out that posttranslational modification of Cnm may facilitate protein translocation or surface localization. We also used surface plasmon resonance (SPR) to characterize the binding of different Cnm fragments to immobilized collagen. Three different truncated constructs (rCnm N_1_, rCnm N_2_, rCnm N_1+2_) (Fig. 4C and D) were designed from the full length (rCnm FL) and used for real time binding analyses with type I collagen. The results from the SPR analysis clearly show that the rcnm N_2_ domain plays a predominant role in adhering to collagen, whilst the rcnm N_1_ by itself does not adhere to collagen (Figs 4E and S3). Notably, the rCnm N_1+2_ fragment displayed a higher K_D_ (∼3-fold higher) value when compared to the rCnm N1 fragment (8.17 × 10^−7^ versus 2.89 × 10^−7^, respectively) suggesting that the N_1_ domain stabilizes the interaction after the initial adherence to the N_2_ domain. Finally, the K_D_ rates for rCnm FL and rCnm N_1+2_ were of a similar order of magnitude. These results are consistent with the known localization of collagen binding activity within the N_2_ domain and serves as further indication that the C-terminal threonine-rich domain predicted to undergo posttranslational modification does not have a direct contribution to the collagen-binding activity of Cnm. While we attempted to study the influence of glycosylation in collagen binding by SPR, we were not able to obtain signals due to the difficulty in purifying a sufficient quantity of native Cnm from *S*. *mutans* OMZ175. Taken together our results suggest that attenuation of Cnm-related phenotypes in the different *pgf* mutants is due primarily to the decrease in Cnm protein stability, rather than a direct influence of the posttranslational modification on Cnm functionality.

### WapA is modified by the Pgf machinery

**Figure 5:**
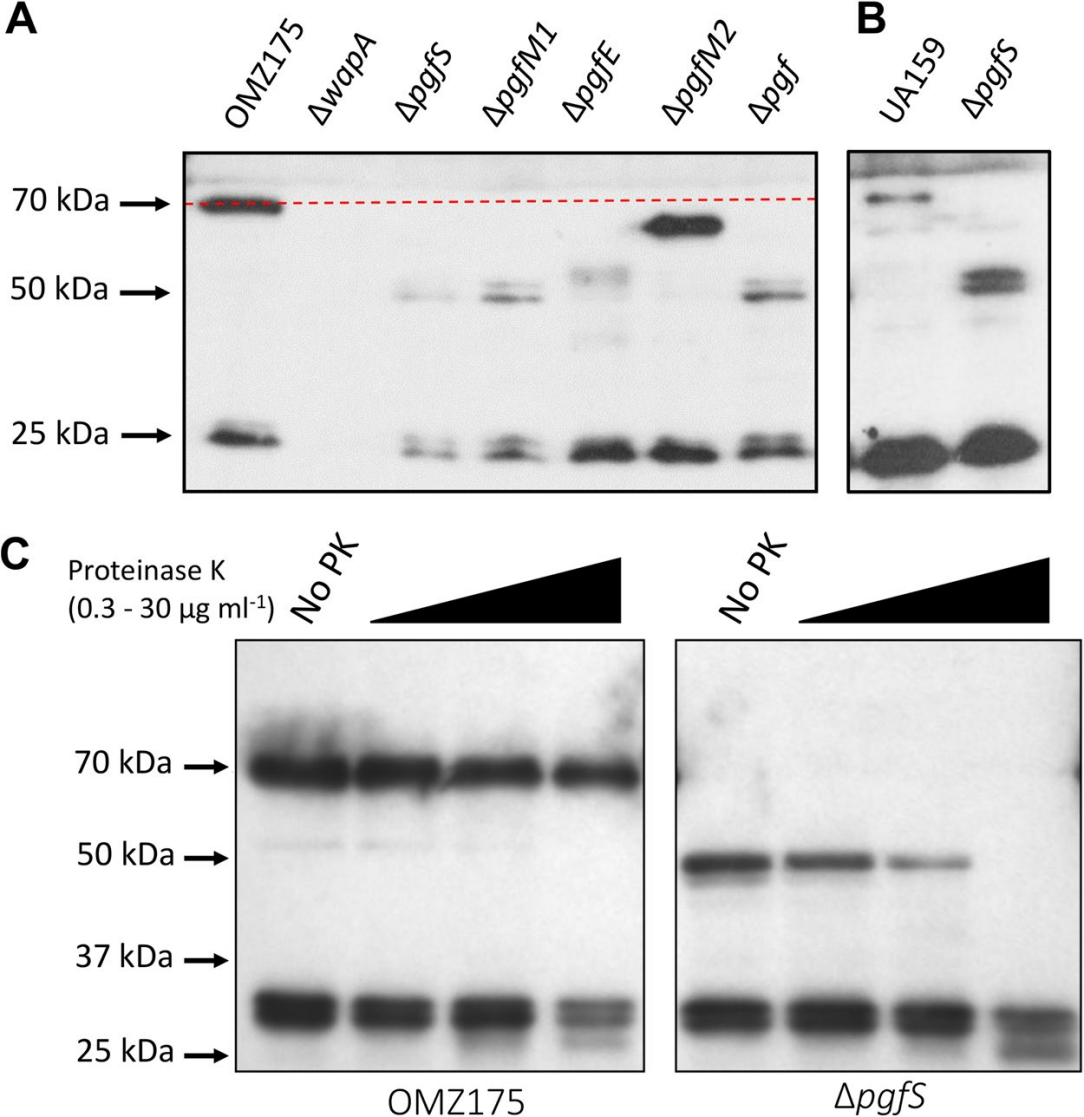
Modification of WapA by the *pgf* operon. (**A**,**B**) Western blot analysis using anti-WapA antibody shows a band of 70 kDa corresponding to the fully mature WapA in OMZ175 (**A**) and UA159 (**B**). Smaller variants of WapA correspond to partially (∼60 kDa Δ*pgfM2*) and unglycosylated WapA (∼50 kDa, Δ*pgfS*, Δ*pgfM1*, Δ*pgfE* and Δ*pgf*). (**C**) Whole cells of strains OMZ175 and Δ*pgfS* derivatives were treated with increasing concentrations of proteinase K for 30 min and WapA stability monitored by Western blotting.

While *cnm* is present in ~15% of *S*. *mutans* strains, *in silico* analysis revealed that the *pgfS*, *pgM1*, *pgfE* and *pgfM2* genes are part of the *S*. *mutans* core genome. We suspected, therefore, that additional proteins encoded by the *S*. *mutans* core genome might be subjected to Pgf-dependent modification. Using the NetOGlyc 4.0 glycoprotein prediction server (http://www.cbs.dtu.dk/services/NetOGlyc/), we identified a number of surface-localized and secreted proteins encoded by *S*. *mutans* core genes that are predicted to undergo *O*-glycosylation (Table 1). We focused on the WapA (wall-associated protein A) adhesin, which has been shown in previous studies to mediate collagen binding *in vitro*, to contribute to sucrose-independent biofilm formation, and to serve as a target of immune protection^26–28^. Of note, the predicted amino acid sequence of WapA is 100% conserved between UA159, a Cnm^−^ non-invasive serotype *c* strain, and OMZ175 (Cnm^+^, serotype *f*) strains. While the predicted size of full-length WapA is ~45 kDa, Western blot analysis of WapA produced by the OMZ175 and UA159 strains revealed protein bands migrating at ~70 kDa, presumably corresponding to a modified form of WapA. A smaller band of ~29 kDa was also observed corresponding to the previously described proteolytic cleavage of WapA at amino acid residues 330 to 340 known as Antigen A or Antigen III^29,30^ (Fig. 5A). Consistent with the notion that the 70 kDa band represents a modified form of WapA, this band was not present in lysates of Δ*pgfS* and Δ*pgfM1* that instead demonstrated a band, sometimes as a doublet, migrating close to the predicted molecular weight of WapA (~45 kDa). Interestingly, the band observed for the Δ*pgfE* mutant was slightly larger those of the Δ*pgfS* and Δ*pgfM1 mutants* while the WapA produced by Δ*pgfM2* was larger than those of the other *pfg* mutants but still smaller than that produced by the parent OMZ175 strain (Fig. 5A). Similar results were observed in a *pgfS* deletion mutant created in the UA159 background strain indicating that the Pgf-dependent modification of WapA is conserved across different *S*. *mutans* strains (Fig. 5B). Finally, migration of the extracellular Antigen A/Antigen III cleavage product of WapA^29,30^ was not affected by deletion of genes encoding *S*. *mutans* Pgf glycosylation machinery components. The amino acids predicted to undergo *O*-glycosylation in WapA are located immediately downstream of the proteolytic cleavage site reported to result in the generation of Antigen A/Antigen III^29^.

**Table 1:** Surface-associated proteins of *S*. *mutans* predicted to undergo *O*-glycosylation.

Gene	Protein (aa)	Predicted glycosylated amino acids
SMU_987	WapA (454)	^343^TTTVTETTTSSSSETTTSEATTETSSTTNNNSTTTETATSTTGASTTQTKTTASQTNVPTTTNITTT^409^
SMU_1005	GtfC (1456)	^67^TATDTSTATSATSQPTATVTDNVSTTNQSTNTTANTANFDVKPTTT^112^
SMU_610	SpaP (1563)	^833^TPPVKPTAPTKPTYETEKPLKPAPVAPNYEKEPTPPTRTPDQAEPNKPTPPTYETEKPLEPAPVEPSYEAEPTPPTRTPDQAEPNKPTPPTYETEKPLEPAPVEPSYEAEPTPPTPTPDQPEPNKPVEPTYEVIPTPPT…TTTPEDPTDPTDPQDPSSPRTST^1509^
SMU_963c	**(299)	^39^TATKSRTTTTTVTSITKSSHQKKEKTNSKWAKQDQP^74^
SMU_648	PrsA (334)	^302^SSGSTTTTTAASSAATTAADDQTTAAETT^330^
SMU_1396	GbpC (584)	^37^TVAAPTADTQASEPAATEKEQSPVVAVVESHT…TPPEKPELKKPTVTWHKNLVVETKTEEVPPVTPPTTPDEPTPEKPKT^515^
SMU_22	GbpB (432)	^304^STTATEAQPSASSASTAAVAANT^326^
SMU_1091	WapE (508)	^48^TVSQADGDNPEQTTSVQQETAPQQTKTSQSSDATVDSEESATSPSDEQT^96^

*Underlined residues are predicted to undergo *O*-glycosylation.

**Putative deacetylase.

Next, we tested the stability of WapA produced by wild-type OMZ175 and Δ*pgfS* strains upon treatment with increasing concentrations of proteinase K. In agreement with the role of glycosylation in protein stability, the WapA product of the Δ*pgfS* strain was more sensitive to proteinase K degradation than the WapA produced by OMZ175 (Fig. 5C). Taken together, our results demonstrate that WapA and Cnm both serve as substrates of the Pgf system. We conclude, therefore, that the Pgf system can modify both core and non-core gene products in *S*. *mutans*.

## Discussion

Initially thought to be a process unique to eukaryotic organisms, protein glycosylation is now recognized to occur in all three domains of life being the most abundant and complex posttranslational modification in nature^1,2^. Previously, we showed that PgfS, a GT-A type glycosyltransferase encoded by a gene located immediately downstream of *cnm*, is required for the glycosylation of Cnm^6^. In this report, we showed that the PgfS-dependent modification of Cnm is a cooperative multi-protein effort that includes at least two membrane proteins (PgfM1 and PgfM2) and an UDP-4-glucose epimerase (PgfE). However, unlike *cnm*, the *pgfS-M1-E-M2* genes are present in all *S*. *mutans* strains. Here, we also showed that the Pgf system can also modify WapA, a surface-associated protein found to be encoded in the genomes of all *S*. *mutans* strains sequenced to date. Moreover, *pgf* homologs are found in other streptococcal species, usually next to genes coding for surface proteins with collagen-binding domains^6,19^. Thus, Pgf-mediated protein modification seems to be broad in scope and to be responsible for the posttranslational modification, likely through glycosylation, of both core and non-core proteins of *S*. *mutans* and, possibly, proteins of other Streptococci.

**Figure 6:**
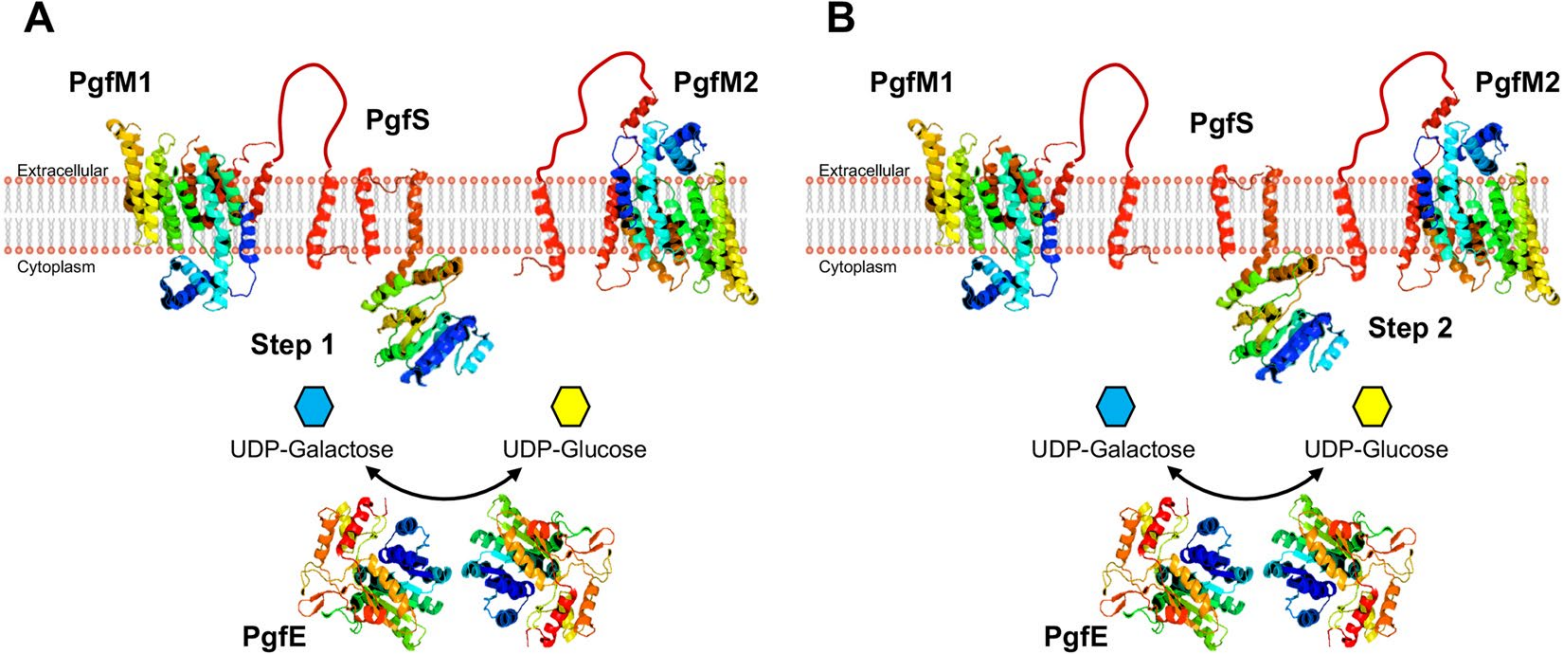
Proposed model of Pgf-mediated protein modification in *S*. *mutans*. Protein localization was predicted using the TMHMM 2.0 software and ribbon structure models were built using the PHYRE software. Both PgfM1 and PgfM2 are integral membrane proteins with an extracellular domain of unknown function. PgfS is membrane-bound with its N-terminal catalytic domain located intracellularly. PgfE is predicted to be intracellular, where it functions as a homodimer to direct the NAD^+^-dependent interconversion of UDP-galactose and UDP-glucose. (**A**) PgfS recognizes the protein target and initiates the process of glycosylation by adding sugars that were initially transported through PgfM1 and subsequently activated by the PgfE sugar epimerase. (**B**) Upon initial transfer, PgfS will add sugars that were transported by PgfM2 and activated by PgfE. Upon completion of the glycosylation process, the mature glycoprotein will be transported to the extracellular milieu and anchored at the cell wall surface via proteolytic cleavage of the C-terminal LPXTG motif.

In *Streptococcus parasanguinis* and *Streptococcus gordonii*, *O*-glycosylation of the fimbrial protein Fap1 and the GspB adhesin, respectively, is stepwise and mediated by several proteins^31–33^. However, in other cases such as in *Acinetobacter baumani*, *O*-glycosylation occurs *en bloc* with the glycan transferred from a lipid carrier to the target protein via *O*-oligosaccharyltransferases (*O*-Otases)^34^. Based on the knowledge that protein glycosylation in Streptococci has been only shown to occur in a sequential manner and that conserved *O*-Otases are absent in *S*. *mutans*, we believe that Cnm and WapA are glycosylated by the Pgf system in a sequencial manner rather than *en bloc*. In support to this possibility, both Cnm and WapA appear to produce a partially gycosylated product in the Δ*pgfM2* background, suggesting that PgfM2 participates in the final steps of both Cnm and WapA glycosylation (Fig. 6). Previously, we showed that the mature (glycosylated) Cnm reacts with WGA and that it specifically recognizes GlcNAc but not sialic acid^6^. The loss of Cnm recognition by WGA in the Δ*pgfM2* mutant suggests that Cnm is probably modified with other sugars that are not recognized by this lectin prior to the addition to GlcNAc. Based on current evidence, we propose that PgfM2 coordinates the addition of GlcNAc as a final step in the protein glycosylation pathway. In contrast, loss of *pgfM1* completely abolished glycosylation suggesting that PgfM1 cooperates with PgfS early in the process (Fig. 6). As part of our future efforts, functional and structural studies will be undertaken to conclusively demonstrate that the Pgf machinery add glycans sequentially.

Despite our best efforts, we have been unable to identify the glycan(s) attached to Cnm using mass spectometry (MS) analysis, possibly because of the heavily glycosylated nature of Cnm makes the protein refractory to enzymatic cleavage. Native Cnm purified from *S*. *mutans* OMZ175 was completely resistant to enzymatic deglycosylation using a cocktail containing five major glycosidases (PNGase F, sialidase A, *O*-glycanase, β(1-4)-galactosidase, of β-*N*-acetylglucosaminidase) and three different proteases (trypsin, Glu-C and pepsin) that failed to digest the threonine-rich B region predicted to undergo *O*-glycosylation^6^. While lectin binding analyses have been instrumental in confirming the glycosylated nature of Cnm, our future efforts will be directed toward engineering a Cnm variant that contains a cleavable site within the threonine-rich B-domain. Thus, upon protease digestion, glycopeptides containing far fewer repeats would be released, hopefully enabling glycan analysis. In addition, as the field of glycobiology continues to evolve, novel approaches in MS, nuclear magnetic resonance (NMR), analytical glycoscience and single-molecule force spectroscopy, among others, should also facilitate a more in-depth characterization of Pgf-dependent glycosylation and its substrates.

The discovery of the Pgf system and the identification of homologous systems in other streptococcal species open the possibility of using *S*. *mutans* as a model organism for glycoproteomics research. This possibility is particularly attractive considering that *S*. *mutans* is a genetically amenable organism with a large number of fast and reliable genetic tools available^35^. Thus, defining the individual functions of PgfS, PgfM1, PgfE and PgfM2 and their biochemical requirements during the glycosylation process will be of paramount importance if this machinery is to be employed in heterologous expression systems and/or in therapeutic targeting of glycosylated virulence factors^36,37^. While a thorough analysis of the Cnm glycosylation process has been difficult due to the technical constraints of its highly repetitive threonine-rich domain, the identification of WapA as an additional target of the Pgf system provides an alternate substrate for the characterization of this glycosylation pathway. In fact the magnitude of the band shift on SDS-PAGE, and the number of threonine and serine residues within the protein, suggest that WapA is not as heavily glycosylated as Cnm and therefore may be more amenable to MS analysis.

Adhesion is a critical first step for infection initiation within a host and involves bacterial surface molecules. Many virulence factors associated with initial colonization, and/or invasion of host cells have been demonstrated to undergo posttranslational modification through glycosylation^1^. The importance of protein glycosylation in virulence has been demonstrated in many bacteria including Gram-positive pathogens^1,38–41^. In *S*. *agalactiae*, for example, the *O-*glycosylated serine-rich surface protein Srr1 is an important virulence factor that contributes to adherence to lung epithelial cell lines and to virulence in a rat model of neonatal sepsis^41^. Inactivation of the glycosylation machinery (GtfCDEFGH) impacted the ability of the mutant strains to display full virulence indicating that glycosylation of Srr1 contributes to bacterium-host cell interactions and resistance to proteolysis^41^. Here, we showed that Cnm modification contributes to invasion of host cells and virulence in the *G*. *mellonella* model. Thus our findings also serve to strengthen the growing literature that posttranslational modification through glycosylation of surface proteins plays an important and still underestimated role in streptococcal virulence.

Taken altogether, this study strongly indicates that the *pgfS*-*M1*-*E*-*M2* operon encodes for a glycosylation pathway responsible for the posttranslational modification of at least two *S*. *mutans* surface proteins. We showed that this posttranslational modification increases the proteolytic stability of Cnm and WapA, a desirable trait for surface exposed adhesins that are constantly challenged by host and bacteria-derived proteases. Thus, the Pgf system could represent an attractive new target for the development of therapeutic approaches against *S*. *mutans* and, possibly, other Streptococci. In addition, a better understanding of how the Pgf system functions can potentially lead to the utilization of this system as a new tool in synthetic biology for the modification of recombinant proteins used for therapeutic and non-therapeutic purposes^1,11,36,42^.

## Methods

### Bacterial strains and culture conditions

Strains of *S*. *mutans* used in this study are listed in Table 2. Strains were routinely cultured in brain heart infusion (BHI) medium at 37 °C in a humidified 5% CO_2_ atmosphere. When required, 1 mg ml^−1^ kanamycin or 10 μg ml^−1^ erythromycin was added to BHI broth or plates. Primary HCAEC were purchased form Lonza Laboratories and cultured in endothelial cell basal medium 2 (EBM-2; Lonza) supplemented with EGM-2MV single-use aliquots (Lonza), as suggested by the supplier.

**Table 2:** *S*. *mutans* strains used in this study.

Strains	Relevant genotype	Source
*S*. *mutans*		
OMZ175	*cnm*^+^, wild-type, serotype *f*	Lab stock
OMZ175Δ*cnm*	*cnm*^−^, *pgf*^+^, kan^R^	^18^
OMZ175Δ*pgfS*	*cnm*^+^, *pgfS*^−^, kan^R^	^6^
OMZ175Δ*pgfM1*	*cnm*^+^, *pgfM1*^−^, kan^R^	This study
OMZ175Δ*pgfE*	*cnm*^+^, *pgfE*^−^, kan^R^	This study
OMZ175Δ*pgfM2*	*cnm*^+^, *pgfM2*^−^, kan^R^	This study
OMZ175Δ*pgf*	*cnm*^+^, *pgf*^−^, kan^R^	This study
OMZ175Δ*wapA*	*cnm*^+^, *wapA*^−^ kan^R^	This study
OMZ175C*pgfM1*	complemented *pgfM1*, kan^S^	This study
OMZ175C*pgfE*	complemented *pgfE*, kan^S^	This study
OMZ175C*pgfM2*	complemented *pgfM2*, kan^S^	This study
OMZ175Δ*smu2063*	*cnm*^+^, *smu2063c*^−^, kan^R^	This study
UA159	*cnm*^−^, wild-type, serotype *c*	Lab stock
UA159Δ*pgfS*	*cnm*^−^, *pgfS*^−^, kan^R^	^6^

### Genetic manipulation of *S*. *mutans*

Mutation of *pgfS* (*smu2067c*) in *S*. *mutans* OMZ175 and UA159 was previously described along with its complemented counterparts^6^. The genes *smu2066c* (*pgfM1*), *smu2065c* (*pgfE*) and *smu2064c* (*pgfM2*) were individually replaced with a non-polar kanamycin cassette via allelic exchange. A quadruple mutant was also generated by replacing all *pgf* genes (*pgfS*-*M1*-*E*-*M2*) with the same non-polar kanamycin cassette used to create the single mutant strains. Briefly, *Pst*I sites were introduced to DNA fragments containing the 5′ and 3′ regions of *pgfM1*, *pgfE* or *pgfM2* using the primers listed in Table 3. For the quadruple mutant construction (Δ*pgf*), DNA fragments containing the 5′ region of *pgfS* and 3′ region of *pgfM2* were generated using primers *pgfS* F1 and *pgfM2* R2. Upon amplification, PCR products were digested with *Pst*I and then ligated to a *Pst*I-digested non-polar kanamycin cassette. The *wapA* gene was inactivated in OMZ175 using a non-polar kanamycin cassette. Briefly, *Kpn*I sites were introduced to DNA fragments containing the 5′ and 3′ regions of *wapA* using the primers listed in Table 3. Upon amplification, PCR products were digested with *Kpn*I and then ligated to a *Kpn*I -digested non-polar kanamycin cassette. Transformation of *S*. *mutans* OMZ175 was carried out by growing bacterial cells to an OD_600_ of 0.1 followed by the addition of 250 ng of DNA along with 100 nM of synthetic competence stimulating peptide (CSP)^43^. Cultures were grown for 4 hours and transformants selected on plates containing kanamycin. All mutations were confirmed by PCR and sequencing of the insertion site.

**Table 3:** Primers used in this study.

Primer name	Sequence	Application
*pgfM1*-F1	AGAACAGCACCATGGACGAGGAAG	Inactivation of *pgfM1*
*pgfM1*-R1	GCTTTCTTACTGCAGGATCTTTGG	Inactivation of *pgfM1*
*pgfM1*-F2	TCTGGTTACTGCAGGGAGCGATGG	Inactivation of *pgfM1*
*pgfM1*-R2	TAGCTAATGAGCTCGCAGCAATCG	Inactivation of *pgfM1*
*pgfE*-F1	AAGATTACCTCCATGGCGCAAACG	Inactivation of *pgfE*
*pgfE*-R1	TATCCACACTGCAGAGCTGATAATC	Inactivation of *pgfE*
*pgfE*-F2	AGAGATTACTGCAGAAGACGTTAC	Inactivation of *pgfE*
*pgfE*-R2	AGCTACCGAGAGCTCAGCATTACC	Inactivation of *pgfE*
*pgfM2*-F1	TCAGATAGTGCCATGGATATTGTC	Inactivation of *pgfM2*
*pgfM2*-R1	AGAAGCAACCTGCAGCCGATGACG	Inactivation of *pgfM2*
*pgfM2*-F2	CTGCCTATGTTCTGCAGGGCAGTCG	Inactivation of *pgfM2*
*pgfM2*-R2	CTGATAACGCTGCAGTGAACGATG	Inactivation of *pgfM2*
*smu2063*-F1	TTGCTTTGGCATGCAATGCTAATGG	Inactivation of *smu2063c*
*smu2063*-R2	GTCCCTAAGTCTGCAGGTAAAACGC	Inactivation of *smu2063c*
*smu2063*-F2	CTATCCTAACTGCAGATGGAAGCC	Inactivation of *smu2063c*
*smu2063*-R2	AGCACTGCTCATATGGTAAAATCC	Inactivation of *smu2063c*
wapA-F1	GAATTTTCAAGATTACTAATGG	Inactivation of *wapA*
*wapA-R1*	CTCCTTAGGGTACCTGTAGAATATTTTG	Inactivation of *wapA*
*wapA-F2*	CCATCAACAGGGTACCAAGGAGGC	Inactivation of *wapA*
*wapA-R2*	CTCACGATAACCCCAAT	Inactivation of *wapA*
*pgfS*-*pgfM1-*F	TGGTGTACAGTTGATTTCC	RT-PCR
*pgfS*-*pgfM1-*R	TGTCCAAAGTCAACTGCC	RT-PCR
*pgfM1-*F	CGATGGGCTTGAGTTTTGC	RT-PCR
*pgfM1-*R	TCACCGATTTCTGCCAAC	RT-PCR
*pgfM1*-*pgfE-*F	AGTCAAGACTGATGAGGC	RT-PCR
*pgfM1*-*pgfE-*R	GTCTTTGCCAGTAATCTC	RT-PCR
*pgfE-*F	ACAGTTCAGGTGAGTTAGG	RT-PCR
*pgfE-*R	AGCAATGTCTCCTGCACG	RT-PCR
*pgfE*-*pgfM2*-F	GCTGAGAAAGAGCTGAATTG	RT-PCR
*pgfE*-*pgfM2*-R	GTGAACGATGCGCACGACTG	RT-PCR
*pgfM2-*F	TTGATGAGCGGTGATTATAC	RT-PCR
*smu2063-*R	ACCGCTCAAACCATAGATAG	RT-PCR
rCnm FL-F	ACTAAGGCTCATATGAGTGATGTC	rCnm purification
rCnm FL-R	CAATATCAGTTGGATCCTTTACGGTAA	rCnm purification
rCnm N_1_-F	ATATCCATGGGGAGTGATGTCAGCAAC	rCnm purification
rCnm N_1_-R	CGCGCTCGAGGCCCTGAAAATACAGGT TTTCAGATTTAACGACAGT	rCnm purification
rCnm N_2_-F	ATATCCATGGGGGCTTCAGGGACTACCGGC	rCnm purification
rCnm N_2_-R	CGCGCTCGAGGCCCTGAAAATACAGGT TTTCTCTTCCGTCCACACC	rCnm purification

Genetic complementation of the *pgf* single mutants was achieved by restoring the original sequence at the exact same location as detailed elsewhere^6^. Briefly, a PCR product containing the intact and flanking region of each *pgf* gene was amplified from OMZ175 using primers listed in Table 3. Cultures were grown overnight in chemically defined medium containing 0.5% glucose (CDMG)^44^, diluted 1:20 in fresh CDMG and grown to OD_600_ 0.1. Cultures of Δ*pgfM1*, Δ*pgfE* or Δ*pgfM2* mutants were then transformed with 250 ng of their respective gene fragment in the presence of 100 nM of ComX-inducing peptide (XIP)^45^, along with 50 ng of pCJK96^46^, which confers resistance to erythromycin, and allowed for the selection of naturally competent cells. After growing cells for 4 h, positive transformants were selected on plates containing erythromycin. Resulting colonies were patched on plates containing erythromycin or kanamycin and colonies that grew in erythromycin but not in kanamycin were screened by PCR. Reintroduction of the *pgfM1*, *pgfE* and *pgfM2* genes was confirmed by sequencing each gene and respective flanking regions.

### *In silico* analysis

The Prokaryotic Promoter Predictor software (http://bioinformatics.biol.rug.nl/websoftware/ppp/) was used to determine putative promoters driving transcription of *pgfS*, *pgfM1*, *pgfE* and *pgfM2*, and ARNold software (http://rna.igmors.u-psud.fr/toolbox/arnold/) was used to determine putative transcriptional terminators. The FGENESB bacterial operon and gene prediction software (http://www.softberry.com/berry.phtml?topic = fgenesb&group = programs&subgroup = gfindb) was used to predict the co-transcription of *pgfS*, *pgfM1*, *pgfE* and *pgfM2*.

### RT-PCR analysis

For RT-PCR, RNA was extracted from *S*. *mutans* OMZ175 cultures grown to mid-exponential phase (OD_600_ ~ 0.5) as previously described^47^. cDNA from 0.5 μg of RNA was synthesized using a high-capacity cDNA reverse transcriptase kit containing random primers (Applied Biosystems). Primers specific for coding and intergenic regions of *cnm*, *pgfS*, *pgfM1*, *pgfE* and *pgfM2* (Table 3) were used to determine the transcriptional organization of these genes.

### Western blot analysis

Overnight cultures were used to prepare whole cell protein lysates of *S*. *mutans* OMZ175, UA159 and derivatives. Briefly, cells were suspended in PBS hand homogenized in the presence of 0.1 mm glass beads using a bead-beater (Biospec) in three intervals of 30 seconds. Protein lysates (10 mg ml^−1^) were separated on 10% SDS-PAGE and transferred to polyvinylidene fluoride (PVDF) membranes (Millipore). Cnm detection was performed using rabbit anti-rCnmA polyclonal antibody^19^ diluted 1:2000 in PBS + 0.1% Tween 20. WapA detection was performed using rabbit anti-WapA polyclonal antibody^48^ diluted 1:2000 in PBS + 0.1% Tween 20. To visualize protein bands, HRP-labeled anti-rabbit horseradish peroxidase secondary antibody (Sigma-Aldrich) diluted 1:2000 was used followed by detection with an enhanced chemiluminescent (ECL) Western blotting detection kit (GE Healthcare).

### Proteinase K susceptibility assay

Susceptibility of Cnm and WapA to protease degradation was determined as previously described^6^. Briefly, cells from overnight cultures of the indicated *S*. *mutans* strains were pelleted by centrifugation and resuspended in 1 × PBS pH 7.2 containing increasing amounts of proteinase K (Sigma-Aldrich). After 30 min incubation on ice, protease activity was neutralized by addition of a protease inhibitor cocktail for 5 min (Thermo Scientific). Bacterial cells were washed once with PBS and Cnm stability was analyzed by Western blot.

### Collagen binding and human coronary artery endothelial cells (HCAEC) invasion

*In vitro* assays for collagen binding and HCAEC invasion were performed as previously described^18,19^. Briefly, for collagen binding assays, 100 μl of PBS-washed bacterial suspensions containing approximately 1 × 10^9^ CFU ml^−1^ were added to each well of a microtiter plate containing immobilized type I collagen from rat tail (Sigma-Aldrich). Adherent cells were stained with 0.05% crystal violet (CV) solution and OD_575_ was measured. To determine the contribution of Cnm glycosylation to collagen binding, experiments were performed as described above but bacteria were pre-incubated with either 500 ng of anti-rCnmA rabbit antiserum or with 1 µg of wheat germ agglutinin (WGA) for 30 min. Samples were then washed with PBS and 100 µl of the cell suspensions were then added to corresponding wells. For HCAEC invasion, 1 ml of 2% FBS-EBM-2 medium containing 1 × 10^7^ CFU ml^−1^ of *S*. *mutans* was used to infect HCAEC-containing wells [multiplicity of infection (MOI) of 100:1], for 2 h in the absence of antibiotics followed by 3 h incubation in 1 ml of 2%FBS-EBM-2 medium containing 300 μg ml^−1^ gentamicin and 50 μg ml^−1^ penicillin G to kill extracellular bacteria. After incubation with antibiotics, HCAECs were lysed with 1 ml of sterile water and the mixture of lysed HCAEC and *S*. *mutans* plated onto TSA agar to determine the number of intracellular bacteria. The percentage of invasion for each strain was calculated based on the initial inoculum and the intracellular bacteria recovered from HCAEC lysates. All experiments were performed at least in triplicate. A one-way ANOVA was performed to verify the significance of binding and invasion between parent and mutant strains. *P* values ≤ 0.05 were considered significant.

### Lectin-binding analysis

Whole cell lysates of *S*. *mutans* strains were separated by SDS-PAGE and transferred to a PVDF membrane as described above. After blocking with 5% bovine serum albumin (BSA) for 1 h at room temperature, the membranes were incubated with 20 μg ml^−1^ of biotinylated wheat germ agglutinin (WGA) (Vector Laboratories) in PBS containing 0.5% BSA for 1 h at room temperature. Membranes were washed three times with PBS containing 0.1% Tween 20, followed by incubation with HRP-conjugated streptavidin (Cell Signaling Technology). Bound lectins were visualized using the ECL detection kit (GE Healthcare).

### *Galleria mellonella* infection

For the *G*. *mellonella* infection model, 5 μl aliquots containing 1 × 10^8^ CFU ml^−1^ in sterile saline of overnight-grown cultures of *S*. *mutans* were injected into the hemocoel of each larva via the last left proleg as detailed elsewhere^47^. Larvae injected with heat-inactivated *S*. *mutans* strains (30 min at 80 °C) were used as controls. After injection, larvae were kept in the dark at 37 °C, and survival was recorded at selected intervals. Experiments were performed in triplicate. Kaplan-Meier killing curves were plotted and estimations of differences in survival were compared using the log-rank test. *P* values ≤ 0.05 were considered significant.

### Expression and purification of recombinant Cnm

To generate full-length recombinant Cnm (rCnm-FL), a derivative of the *cnm* gene encoding amino acids 32–465 was amplified from OMZ175 using the primers listed in Table 3. The amino acids for the N-terminal secretion signal (1–31) were excluded to avoid toxic effects in *E*. *coli*. The amplified PCR product and the expression vector pET16b (Clontech) were each digested with *Nde*I and *Bam*HI, ligated and transformed into *E*. *coli* BL21 (DE3). Cells harboring the pET16b::rCnm-FL were grown in LB containing ampicillin to OD_600_ 0.5 and expression was achieved by the addition of 0.5 mM isopropyl-β-D-thiogalactopyranoside (IPTG) for 18 h at 24 °C. Three additional constructs (rcnm N_1_, rcnm N_2_, rcnm N_1+2_) were designed from rCnm FL using primers listed in Table 3. The PCR products and the pET23d expression vector were then digested with *Xho*I and *Nco*I, ligated and transformed into *E*. *coli* BL21 (DE3). Cells bearing the recombinant plasmids were grown to OD_600_ 0.6 and expression was achieved by the addition of 1 mM IPTG at 37 °C for 4 h. For protein purification, cells were harvested by centrifugation, lysed, filtered and then loaded onto His-Prep affinity columns (GE Healthcare). Non-specific proteins were eluted with 50 mM imidazole in binding buffer, while proteins of interest were eluted with a 50–300 mM imidazole gradient of the same buffer. After confirming expression and purification of the different fragments by SDS-PAGE, the respective fractions were pooled and dialyzed against 20 mM HEPES, pH 7.4 for subsequent analysis.

### Surface plasmon resonance

Real-time binding analyses of Cnm proteins (rCnm N_1_, rCnm N_2_ rCnm N_1+2_ and rCnm FL) with type I collagen (BD-Biosciences, Franklin Lakes, NJ) were carried out using the BIAcore 2000 system (GE Healthcare) (Fig. 4C). A CM5 chip was labeled with collagen ligand as previously described^49^, using the amine coupling kit (GE Healthcare) followed by blocking of both control and experimental surfaces using 1 M ethanolamine. Various concentrations (0.125 µM to 2.5 µM) of rCnm analytes were injected over the prepared chip surfaces and dissociation was measured for 8 to 10 min at a flow rate of 20 μl min^−1^ of binding buffer (20 mM HEPES, pH 7.4, 150 mM NaCl, 2.5 mM CaCl_2_) at 25 °C. The regeneration of the surface between experiments was accomplished using 1 M NaCl, 20 mM EDTA, pH 7.2. All experiments were carried out in triplicate and the kinetics of the association (K_A_) and dissociation (K_D_) rate constants were deduced using the 1:1 Langmuir Kinetic model on the BIA-Evaluation software (GE Healthcare).

### Evaluation of cell-surface localized Cnm by flow cytometry

Strains were grown overnight in BHI, diluted 1:40 in fresh medium and grown to OD_600_ of 0.5. Cultures were then washed three times in PBS by centrifugation at 13,000 RPM for 3 min and 500 μl of cells (approximately 1 × 10^8^ cfu ml^−1^) were incubated with 2.5 μg of purified anti-rCnm rabbit IgG in 500 μl PBS for 1 h at room temperature with continuous rotation. Cells were then washed three times in PBS followed by incubation with 5 μg of Alexa-488 anti-rabbit in 500 μl PBS for 1 h at room temperature with continuous rotation. Finally, cells were washed three more times in PBS, resuspended in 500 μl PBS and surface-localized Cnm was quantified using the Accuri C6 (BD-Biosciences) Flow Cytometer at the University of Florida Cytometry Core.

## Electronic supplementary material

Supplemental data
