# Supplementary material for: Characterization of the pgf operon involved in the posttranslational modification of Streptococcus mutans surface proteins
Source: Sci Rep. 2018 Mar 16;8:4705. doi: 10.1038/s41598-018-23170-3 (PMC5856776; doi:10.1038/s41598-018-23170-3)
Supplement: Supplementary file 1 — Supplemental data [file 41598_2018_23170_MOESM1_ESM.pdf]

Characterization of the *pgf* operon involved in the posttranslational modification of *Streptococcus mutans* surface proteins

Alejandro Avilés-Reyes[1], Irlan Almeida Freires[1], Richard Besingi[1], Sangeetha Purushotham[2], Champion Deivanayagam[2], L. Jeannine Brady, Jacqueline Abranches[1*] and José A. Lemos[1*]

[1]Department of Oral Biology, University of Florida, College of Dentistry, Gainesville, FL
[2]Department of Biochemistry and Molecular Genetics, University of Alabama, Birmingham, AL

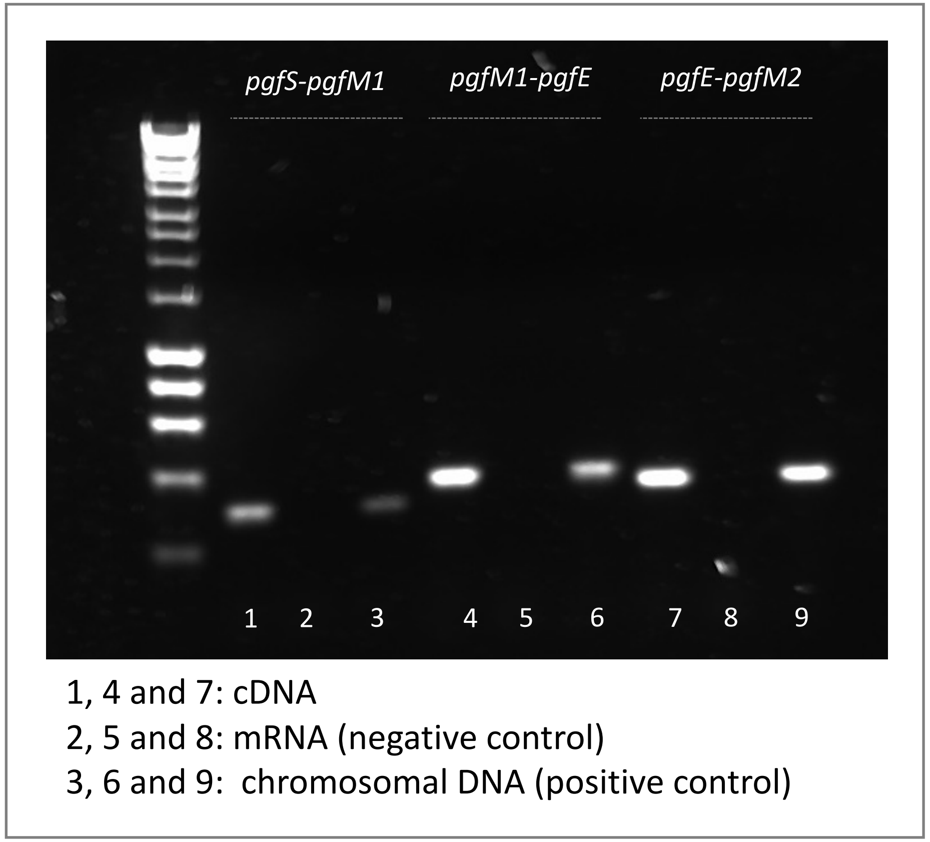

Figure S1. PCR products generated by RT-PCR. Shown is an ethidium bromide-stained agarose gel of products obtained with primers spanning the intergenic regions between *pgfS-pgfM1* (lanes 1 to 3), *pgfM1-pgfE* (lanes 4 to 6) and *pgfE-pgfM2* (lanes 7 to 9). Products from the PCR were derived with the following: cDNA prepared from *S. mutans* OMZ175 mRNA (lanes 1, 4 and 7), a negative control using mRNA but omitting RT (lanes 2, 5 and 8), and a positive chromosomal DNA control (lanes 3, 6 and 9).

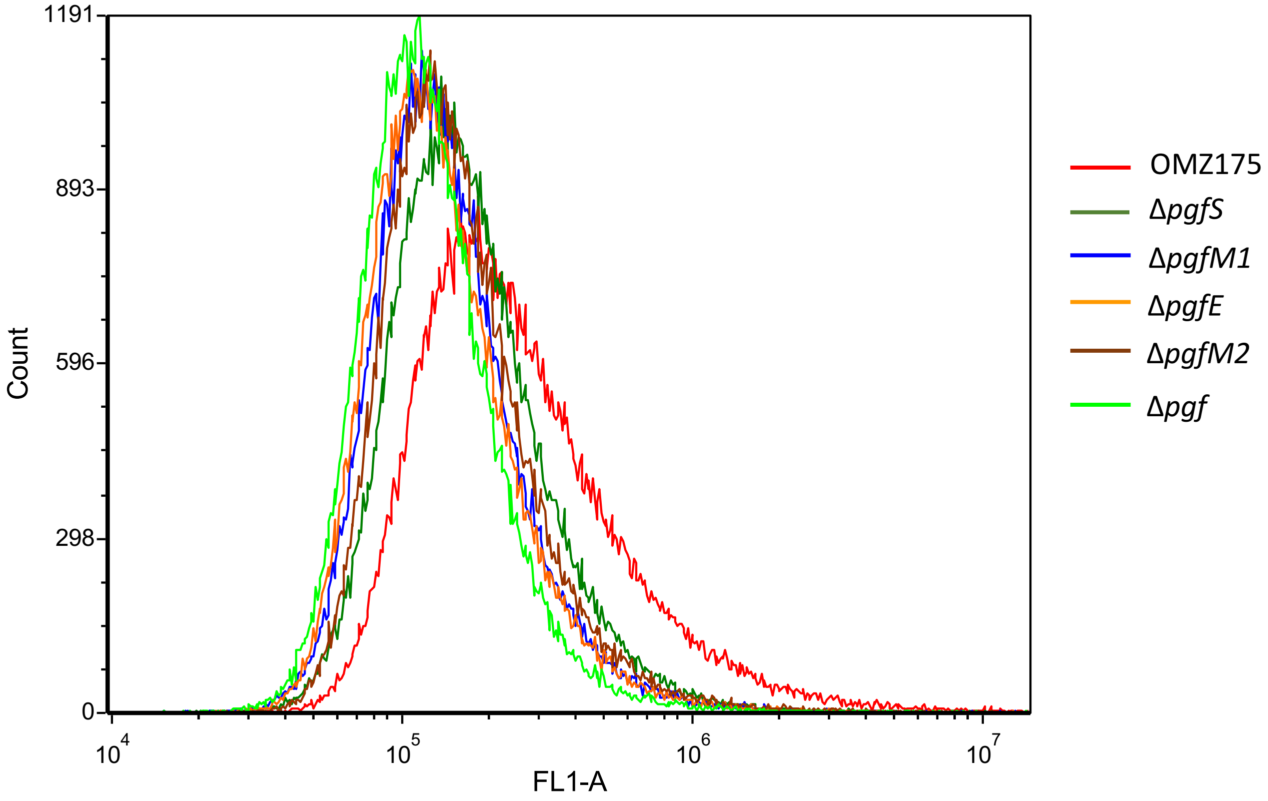

Figure S2. Representative histograms of expression of Cnm on the surface of OMZ175 and its Δ*pgf* derivatives by flow cytometry. Refer to Figure 4 and methods section for additional details.

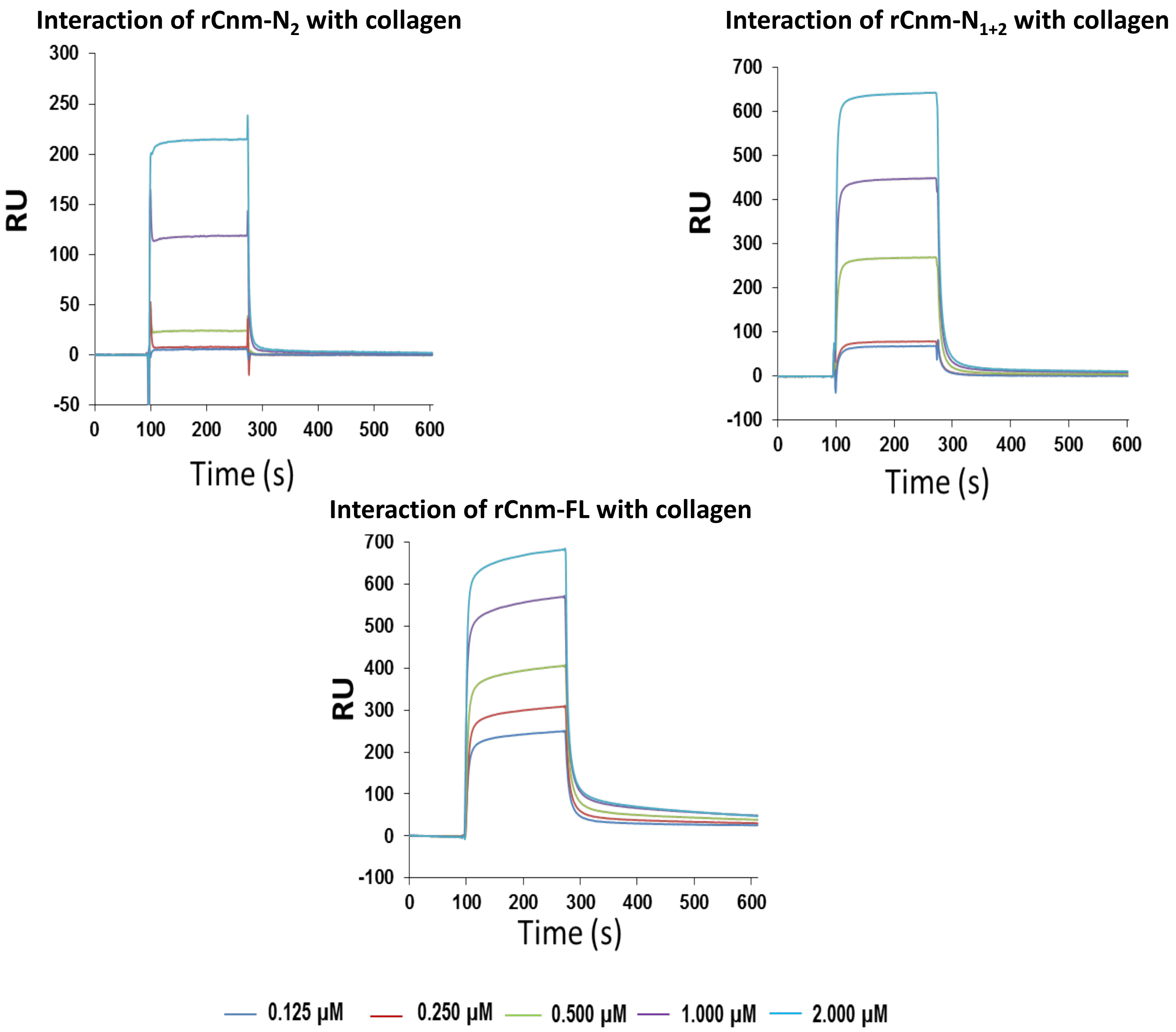

Figure S3. Surface Plasmon Resonance (SPR) sensograms of binding of different Cnm fragments to immobilized collagen. Refer to Figure 4 and methods section for additional details.
